# CyCadas: accelerating interactive annotation and analysis of clustered cytometry data

**DOI:** 10.1093/bioinformatics/btae595

**Published:** 2024-10-07

**Authors:** Oliver Hunewald, Agnieszka Demczuk, Joseph Longworth, Markus Ollert

**Affiliations:** Department of Infection and Immunity, Luxembourg Institute of Health, L-4354 Esch-sur-Alzette, Luxembourg; Bioinformatics & AI, Department of Medical Informatics, Luxembourg Institute of Health, L-1445 Strassen, Luxembourg; Department of Infection and Immunity, Luxembourg Institute of Health, L-4354 Esch-sur-Alzette, Luxembourg; Faculty of Science, Technology and Medicine, University of Luxembourg, L-4365 Esch-sur-Alzette, Luxembourg; Department of Infection and Immunity, Luxembourg Institute of Health, L-4354 Esch-sur-Alzette, Luxembourg; Immunology & Genetics, Luxembourg Centre for Systems Biomedicine (LCSB), University of Luxembourg, L-4362 Esch-sur-Alzette, Luxembourg; Department of Infection and Immunity, Luxembourg Institute of Health, L-4354 Esch-sur-Alzette, Luxembourg; Department of Dermatology and Allergy Centre, Odense University Hospital, 5000 Odense, Denmark

## Abstract

**Motivation:**

Single cell profiling by cytometry has emerged as a key technology in biology, immunology and clinical-translational medicine. The correct annotation, which refers to the identification of clusters as specific cell populations based on their marker expression, of clustered high-dimensional cytometry data, is a critical step of the analysis. Its accuracy determines the correct interpretation of the biological data. Despite the progress in various clustering algorithms, the annotation of clustered data still remains a manual, time consuming and error-prone task. We developed a user-friendly cluster annotation and differential abundance detection tool that can be applied on data generated with Self Organizing Map clustering algorithms, thus simplifying the annotation process of datasets that consist of hundreds or thousands of clusters.

**Results:**

We present Cytometry Cluster Annotation and Differential Abundance Suite (CyCadas), a semi-automated software tool that facilitates cluster annotation in cytometry data by offering both visual and computational guidance. CyCadas addresses the critical need for efficient and accurate annotation of high-resolution clustered cytometry data, significantly reducing the time needed to perform the analysis compared to both manual gating approaches and manual annotation of clustered data. The tool features a user-friendly interface, visual tools enabling data exploration and automated threshold estimation to separate negative and positive marker expression. It facilitates the definition and annotation of cell phenotypes among multiple clusters in a tree-based data structure. Finally, it calculates the abundance of various cell populations across the conditions with statistical interpretation. It is an ideal resource for researchers aiming to streamline their cytometry workflow.

**Availability and implementation:**

CyCadas is available as open source at: https://github.com/DII-LIH-Luxembourg/cycadas.

## 1 Introduction

Current developments in the field of mass and spectral flow cytometry, as well as conducting large-scale longitudinal clinical studies result in generation of huge datasets, analysis of which is mostly performed by unsupervised clustering and dimension reduction algorithms—SPADE (Zaki 2001), FlowSOM (Van Gassen *et al.* 2015), GigaSOM (Kratochvíl *et al.* 2020), PhenoGraph (Levine *et al.* 2015), ClusterX (Chen *et al.* 2016), FlowGrid (Ye and Ho 2019), among others. Furthermore, several pipelines such as CATALYST (Crowell *et al.* 2024), CYANUS (Arend *et al.* 2022), and ImmunoCluster (Opzoomer *et al.* 2021) enable clustering and differential abundance analysis. These tools have been developed to reduce the time required for analysis and minimize the risk of bias that researchers may introduce through manual gating.

However, the annotation of clusters as biologically relevant cell types remains a manual task (Behbehani *et al.* 2020, Vanderbeke *et al.* 2021, Lei *et al.* 2023, Mulhearn *et al.* 2023, Nishide *et al.* 2023, Nuñez *et al.* 2023). This process is especially time-consuming and error-prone when targeting small cell subsets, as clustering needs to be performed with high resolution (over-clustering) and clusters are subsequently merged by similarity (Nowicka *et al.* 2017). An alternative approach proposed by Weber and colleagues consists in over-clustering and inspecting the phenotypes of differentially abundant clusters only. This might greatly reduce the overall time needed for the annotation process, but often results in the loss of statistical power when testing is performed on individual clusters due to multiple testing penalty. Nevertheless, identification and merging of these clusters as cell populations is still a manual process (Weber *et al.* 2019). Furthermore, to assess the robustness of clustering, it should be repeated multiple times with different random seeds (Nowicka *et al.* 2017). Multiple processing inflates further the time consumption for annotation and increases the potential for inconsistencies between annotations.

To address the need for efficient and reproducible annotation of even large numbers of clusters generated with any Self Organizing Map (SOM)-based clustering algorithms, we developed **Cy**tometry Cluster Annotation and Differential Abundance Suite (CyCadas)—an R-based software tool utilizing the Shiny framework supporting an interactive and user-friendly browser-based graphical interface that facilitates the exploration, visualization, annotation and differential abundance analysis of clustered data. CyCadas provides a semi-automated visual cluster exploratory solution, enabling users to easily identify and classify different cell types within their dataset. Although CyCadas is able to annotate small scale datasets, this software tool has been designed to streamline the cluster annotation process within a high-resolution cytometry data analysis workflow and is particularly useful in the analysis of data clustered with a high performance computing (HPC) workflow, as it can rapidly annotate hundreds or thousands of clusters in a straightforward way.

## 2 Functionality and usage

**Figure 1.**
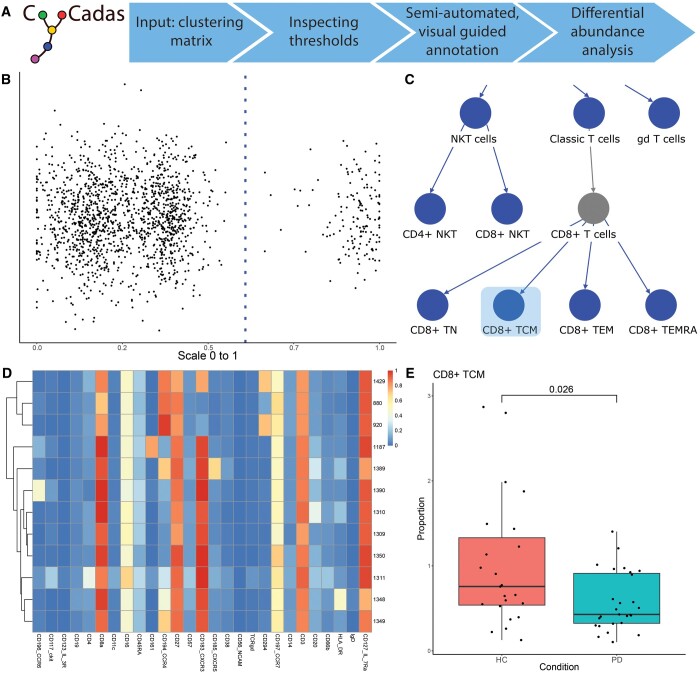
Overview of CyCadas functionalities. (A) CyCadas workflow. (B) Scatter plot representing cluster distribution based on the normalized expression of selected marker. The dotted line designates threshold value defined by the k-means clustering. (C) Cropped fragment of the annotation tree representing the hierarchy of identified subpopulations. An emptied node (example shown: CD8+ T cells) changes color. The CD8+ TCM population is highlighted in a box, its phenotype and differential abundance between the conditions can be inspected on subfigures D and E, respectively. (D) Heatmap visualizing marker expression across selected clusters (defined by the set of negative and positive markers). (E) Boxplot representing abundance of selected cell type across the conditions. HC, healthy control; PD, Parkinson’s disease (refer to Section 2.4 for further details).

At the initial stage (Fig. 1A), the data required for annotation can be provided in individual comma-separated values (CSV) file format (*.CSV files; expression data, cluster frequency, and optionally metadata and cluster counts of each sample if differential abundance analysis is desired). When utilizing R-based software packages (e.g. CATALYST or CYANUS), the CyCadas data can be easily exported in R data serialization (RDS) file format (*.RDS file).

Detailed coding examples enabling the extraction of required data from a typical unsupervised clustering algorithm such as FlowSOM (R) and GigaSOM (Julia), as well as instructions for saving the Single Cell Experiment (SCE) object files from CATALYST and CYANUS can be found in the online manual (https://github.com/DII-LIH-Luxembourg/cycadas).

### 2.1 Marker expression

The core functionality of CyCadas relies on the separation of high/positive and low/negative expression for each marker, typically exhibiting a bimodal distribution in mass cytometry. This threshold value is calculated using two methods: 1D k-means clustering and Gaussian mixture model (GMM). The performance of these methods is then assessed by silhouette analysis (Rousseeuw 1987), which determines the separation between the clusters. The threshold generated by the method that obtained a higher silhouette score is used for the annotation and is indicated by a vertical line within a scatter plot (Fig. 1B) and histogram. Furthermore, the bimodality for every marker is assessed using the R package “mousetrap” (Wulff *et al.* 2023) and the bimodal coefficient values are reported besides the estimated threshold values. If the data meets the bimodal distribution criteria [coefficient value < 0.555 (Pfister *et al.* 2013)], the vertical line is coloured in blue, otherwise it is shown in red (Supplementary Fig. S1). In case the markers are not fulfilling the bimodality criteria, the users are advised to consider the following steps:(i) increasing the clustering resolution (i.e. performing the analysis with a higher number of clusters), (ii) excluding these markers from the phenotype analysis, and (iii) manually adjusting the threshold value by clicking on the scatterplot.

Regardless of the results from the bimodality tests, any threshold should be considered as an estimation and users are advised to carefully inspect each marker distribution and its threshold value. Adjustments of the threshold values, as well as the inclusion of markers into the annotation process, which do not fulfil the bimodal criteria, should be taken with care and require validation by scientists with expertise in cytometry. The threshold values can be saved in a CSV file format and re-loaded into CyCadas. This enables the reproducibility and continuation of the analysis at any time as well as collaborative efforts among individual researchers and across research groups.

### 2.2 Annotation

The annotation follows a tree-based hierarchical process that starts by defining main cell types and subsequently their subtypes. Initially, all clusters are belonging to the root node, and are referred to as “unassigned.” The user then specifies a set of positive and/or negative markers to identify a population of interest as a child node. Clusters are then automatically filtered according to their marker selection into a child node. Importantly, if any marker expression threshold is altered, the tree is automatically recalculated and updated. Consequently, the list of defining antigen markers within a subtype can be viewed as the cumulative markers within its branch—for instance, CD8+ T cells are characterized by the following expression pattern: CD66b− (non-granulocytes) → CD3+CD19− (T cells) → CD56−TCRgd− (classic T cells) → CD8+CD4− (CD8+ T cells), and expression of CCR7+CD45RA− defines central memory CD8 T cells (CD8+ TCM) (Fig. 1C). The user can select multiple positive and negative markers to define the phenotype of the population of interest. An example annotation of CD8+ T cell subsets using nine markers in parallel is shown in the Supplementary Material (Supplementary Fig. S2). The full panel of cell type annotation definitions is provided in the Supplementary Material (Supplementary Table S1).

If all clusters in a parent node are assigned to their respective child nodes, the parent node is coloured in grey, indicating that it is fully defined by its child nodes without any remaining unattributed clusters (Fig. 1C). The expression pattern of the clusters within a selected node is visualized in a heat map (Fig. 1D depicts CD8+ TCM cells selected in Fig. 1C). In addition, these clusters are highlighted in a Uniform Manifold Approximation and Projection (UMAP) plot (Supplementary Fig. S3). The cluster counts and percentages of a selected node are reported in a text field.

The cluster annotation can be saved as CSV file and reloaded, facilitating the reprocessing of the data and continuation of the analysis at any time. It enables applying the same annotation strategy to data generated in multiple clustering rounds with different random seeds, as well as to other datasets with identical phenotypical marker selections.

### 2.3 Differential abundance analysis and data visualization

When performing differential abundance analysis, cluster counts with identical names are summed and the annotated proportion for each sample is calculated. A pairwise Wilcoxon test (using pairwise.wilcox.test function) is performed, allowing the user the choice of the adjustment method for multiple testing (e.g. Hochberg, Bonferroni, or FDR). As this test will be performed on every individual node, remaining clusters in a parent node will be treated as individual phenotypes and renamed accordingly, e.g. “NKT cells” → “NKT cells_remaining.” Additionally, the differential abundance of an individual phenotype can be explored in an interactive tree by selecting a single node in the graph. This aggregates all child nodes (sub-phenotypes) into its selected parent phenotype. An example boxplot generated upon selecting CD8+ TCM cells is shown in Fig. 1E. The table containing the percentage of each identified population in each sample can be exported and used for more advanced statistical interpretation.

### 2.4 Demo dataset

In order to facilitate the tool exploration, we provide a demo dataset that can be used for testing purposes. It can be loaded as annotated data (including the example annotation tree) or as cluster expression data which allows the user to create their own annotation. This demo dataset is generated from the publicly available mass cytometry data of patients with idiopathic Parkinson’s disease (PD) and healthy controls (HC) (Capelle *et al.* 2023a,b), where the data analysis was performed by manual gating using the FlowJo™ Software. Whole blood cell samples stained with a mass cytometry panel as detailed in the original article (Capelle *et al*. 2023b, see Supplementary Table S3) were clustered with GigaSOM to generate 1600 clusters.

Unsupervised clustering of the PD and HC dataset and CyCadas-based annotation allowed us to essentially reproduce the main findings of the original study. CyCadas-generated plots showing the frequency of selected cell populations are shown in Supplementary Fig. S4. These results are highly compatible with the frequencies acquired by manual annotation as reported in the original article ([Bibr btae595-B4][Bibr btae595-B4]).

### 2.5 Implementation and availability

CyCadas is implemented using the R programming language and its user interface is built using the Shiny framework. It is open-source and available for download from the Github repository. A detailed description of every function is available as online manual (https://github.com/DII-LIH-Luxembourg/cycadas).

## 3 Conclusion

In this work, we present CyCadas—a user-friendly solution for cytometry cluster exploration and analysis, enabling researchers to gain deep insights into the cellular heterogeneity of the data. This semi-automated, visually guided annotation software can greatly enhance the efficiency and accuracy of cell clustering analysis.

CyCadas allows users to visualize marker expression patterns, easily identify distinct cell populations, annotate clusters as specific cell types based on the biological knowledge, and perform differential abundance analysis. By incorporating both visual and analytical approaches, this tool can help to reduce user bias, improve reproducibility and save considerable time in comparison to manual annotation methods. This allows the analysis of even huge datasets, such as the provided demo dataset of PD patients and HCs (Capelle *et al.*2023a,b), within several hours to a few days, depending on the complexity of the project. Annotation data from CyCadas can be exported and re-loaded, thus enabling consistent, fast and reproducible re-analysis of the data, as well as a clear method deposition for FAIR data compliance with the possibility to apply strictly the same annotation strategy over multiple clustered datasets.

CyCadas’ utilization of a Shiny-based framework minimizes the access requirements for the standard biological investigator. It is an interactive tool that can greatly simplify collaboration between biologists and bioinformaticians, contributing to improved reproducibility in research.

## Supplementary Material

btae595_Supplementary_Data

## Data Availability

The data (CyCadas code and demo dataset) are available in Github repository at https://github.com/DII-LIH-Luxembourg/cycadas.
