# Supplementary material for: CyCadas: accelerating interactive annotation and analysis of clustered cytometry data
Source: Bioinformatics. 2024 Oct 7;40(10):btae595. doi: 10.1093/bioinformatics/btae595 (PMC11488975; doi:10.1093/bioinformatics/btae595)
Supplement: btae595_Supplementary_Data [file btae595_supplementary_data.zip › CyCadas_supplement.docx]

**CyCadas: Accelerating interactive annotation and analysis of clustered cytometry data – Supplementary Material**


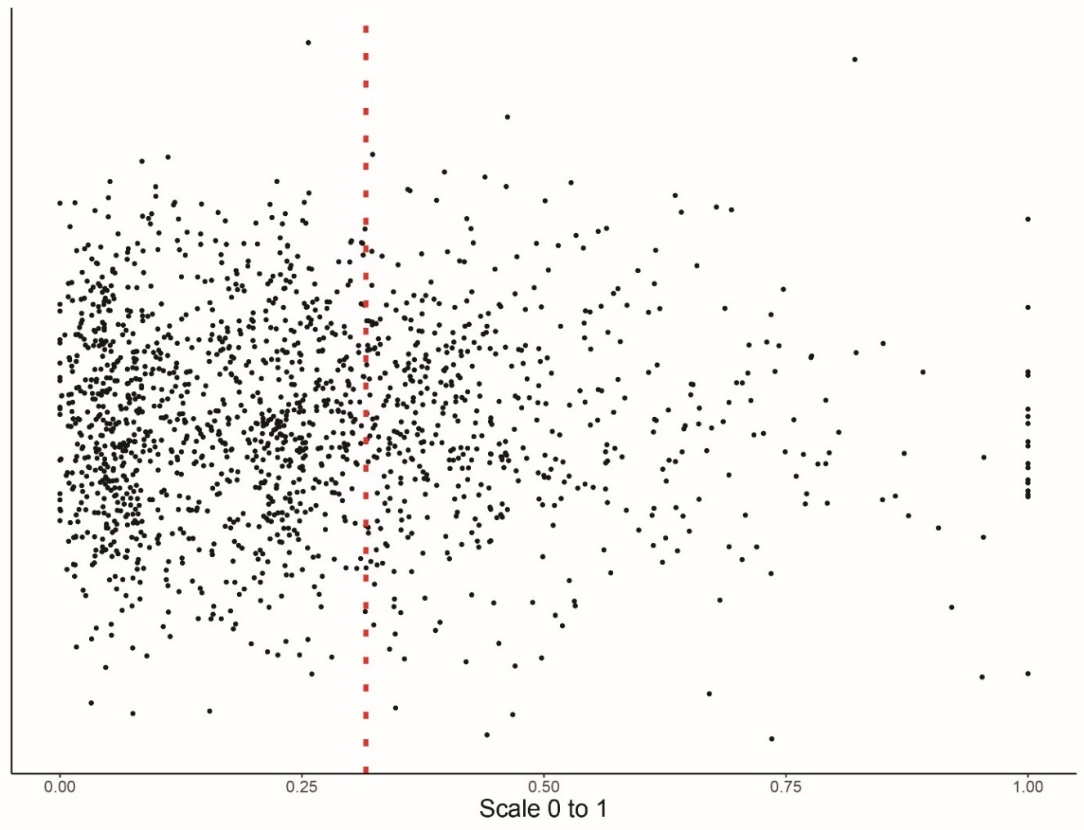


Figure S1 Scatter plot depicting marker expression distribution with generated threshold indicated by the red line due to non-bimodal distribution. Threshold value can be manually adjusted by the user.


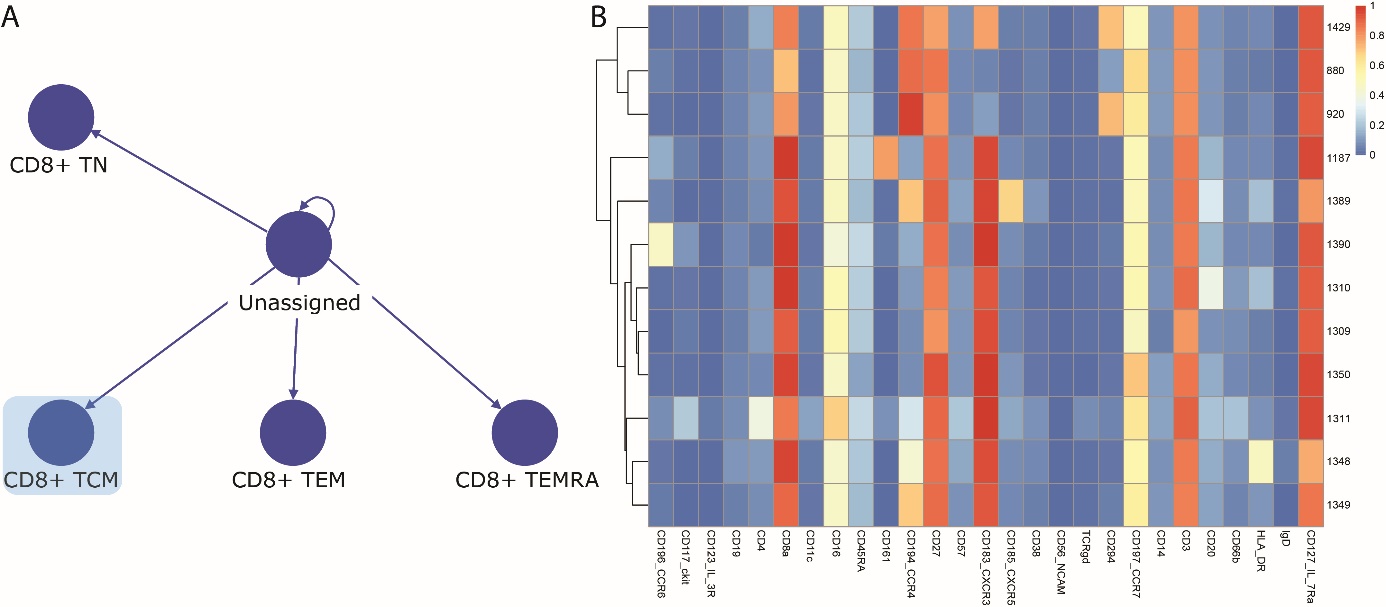


Figure S2 Example annotation of CD8+ T cell subsets using 9 markers in parallel (CD3, CD8, CD66b, CD19, CD56, TCRgd, CD4, CCR7 and CD45). A. Cropped fragment of the annotation tree. CD8+ TCM population is highlighted in blue. B. Heatmap displaying the phenotype of clusters identified as CD8+ TCM cells.


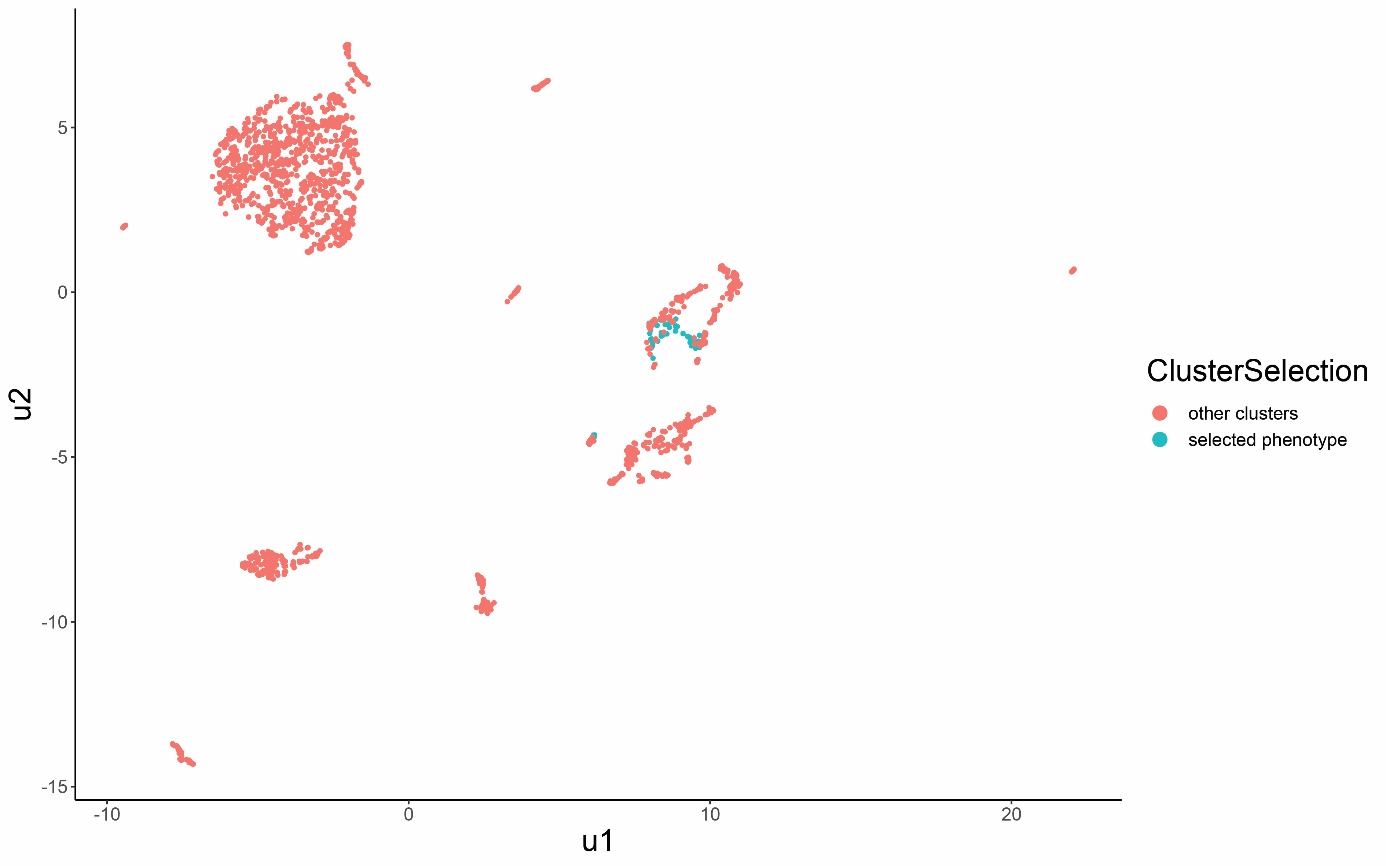


Figure S3 UMAP plot depicting the selected population highlighted in blue.


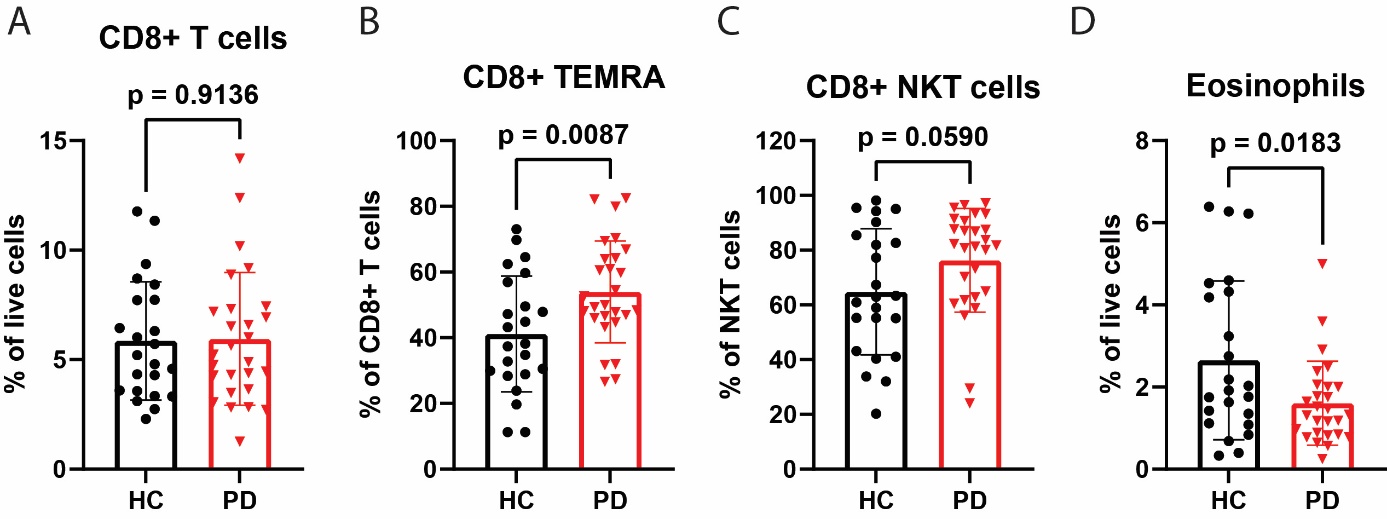


Figure S4 Results of the annotation of Parkinson’s disease study dataset (Capelle, et al., 2023) with CyCadas. A-D. Scatter dot plots showing the frequency of selected cell populations that were identified in the referenced study (Figure 1d, 1e, 1h, 1o).

| **Population** | **Phenotype** |
| --- | --- |
| Eosinophils | CD66b+ CD294+ CD16- |
| Neutrophils | CD66b+ CD294- CD16+ |
| Basophils | CD66b- CD123+ CD38+ CD19- CD3- CD56- HLA-DR- |
| B cells | CD66b- CD19+ CD3- |
| Plasma cells | CD66b- CD19+ CD3- HLA-DR+ CD27+ CD38+ CD20- |
| Naïve B cells | CD66b- CD19+ CD3- HLA-DR+ CD20+ IgD+ CD27- |
| Class-switched memory B cell | CD66b- CD19+ CD3- HLA-DR+ CD20+ IgD- CD27+ |
| IgM memory B cells | CD66b- CD19+ CD3- HLA-DR+ CD20+ IgD+ CD27+ |
| Non-class and interm Mono | CD66b- HLA-DR+ CD3- CD19- CD38- |
| classical Mono | CD66b- HLA-DR+ CD3- CD19- CD14+ CD38+ |
| mDCs | CD66b- HLA-DR+ CD3- CD19- CD14- CD38+ CD11c+ CD123- |
| pDCs | CD66b- HLA-DR+ CD3- CD19- CD14- CD38+ CD11c- CD123+ |
| NK cells | CD66b- CD56+ CD19- CD3- HLA-DR- |
| NKT cells | CD66b- CD3+ CD19- CD56+ TCRgd- |
| CD4+ NKT cells | CD66b- CD3+ CD19- CD56+ TCRgd- CD4+ CD8- |
| CD8+ NKT cells | CD66b- CD3+ CD19- CD56+ TCRgd- CD4- CD8+ |
| gd T cells | CD66b- CD3+ CD19- CD56- TCRgd+ |
| classic T cells | CD66b- CD3+ CD19- CD56- TCRgd- |
| CD8+ T cells | CD66b- CD3+ CD19- CD56- TCRgd- CD4- CD8+ |
| CD8+ TN | CD66b- CD3+ CD19- CD56- TCRgd- CD4- CD8+ CCR7+ CD45RA+ |
| CD8+ TCM | CD66b- CD3+ CD19- CD56- TCRgd- CD4- CD8+ CCR7+ CD45RA- |
| CD8+ TEM | CD66b- CD3+ CD19- CD56- TCRgd- CD4- CD8+ CCR7- CD45RA- |
| CD8+ TEMRA | CD66b- CD3+ CD19- CD56- TCRgd- CD4- CD8+ CCR7- CD45RA+ |
| CD4+ T cells | CD66b- CD3+ CD19- CD56- TCRgd- CD4+ CD8- |
| CD4+ TN | CD66b- CD3+ CD19- CD56- TCRgd- CD4+ CD8- CCR7+ CD45RA+ |
| CD4+ TCM | CD66b- CD3+ CD19- CD56- TCRgd- CD4+ CD8- CCR7+ CD45RA- |
| CD4+ TEM | CD66b- CD3+ CD19- CD56- TCRgd- CD4+ CD8- CCR7- CD45RA- |
| CD4+ TEMRA | CD66b- CD3+ CD19- CD56- TCRgd- CD4+ CD8- CCR7- CD45RA+ |
| Th1 | CD66b- CD3+ CD19- CD56- TCRgd- CD4+ CD8- CD45RA- CXCR5- CXCR3+ CCR4- CCR6- |
| Th2 | CD66b- CD3+ CD19- CD56- TCRgd- CD4+ CD8- CD45RA- CXCR5- CXCR3- CCR4+ CCR6- |
| Th17 | CD66b- CD3+ CD19- CD56- TCRgd- CD4+ CD8- CD45RA- CXCR5- CXCR3- CCR4+ CCR6+ |
| Tfh | CD66b- CD3+ CD19- CD56- TCRgd- CD4+ CD8- CD45RA- CXCR5+ |

Table S1 Phenotypes (positive and negative markers) of defined populations.

References:

Capelle, C.M.*, et al.* Early-to-mid stage idiopathic Parkinson’s disease shows enhanced cytotoxicity and differentiation in CD8 T-cells in females. *Nat Commun* 2023;14(1):7461.
