# Supplementary figures and images for: CyCadas: accelerating interactive annotation and analysis of clustered cytometry data
Source: Bioinformatics. 2024 Oct 7;40(10):btae595. doi: 10.1093/bioinformatics/btae595 (PMC11488975; doi:10.1093/bioinformatics/btae595)

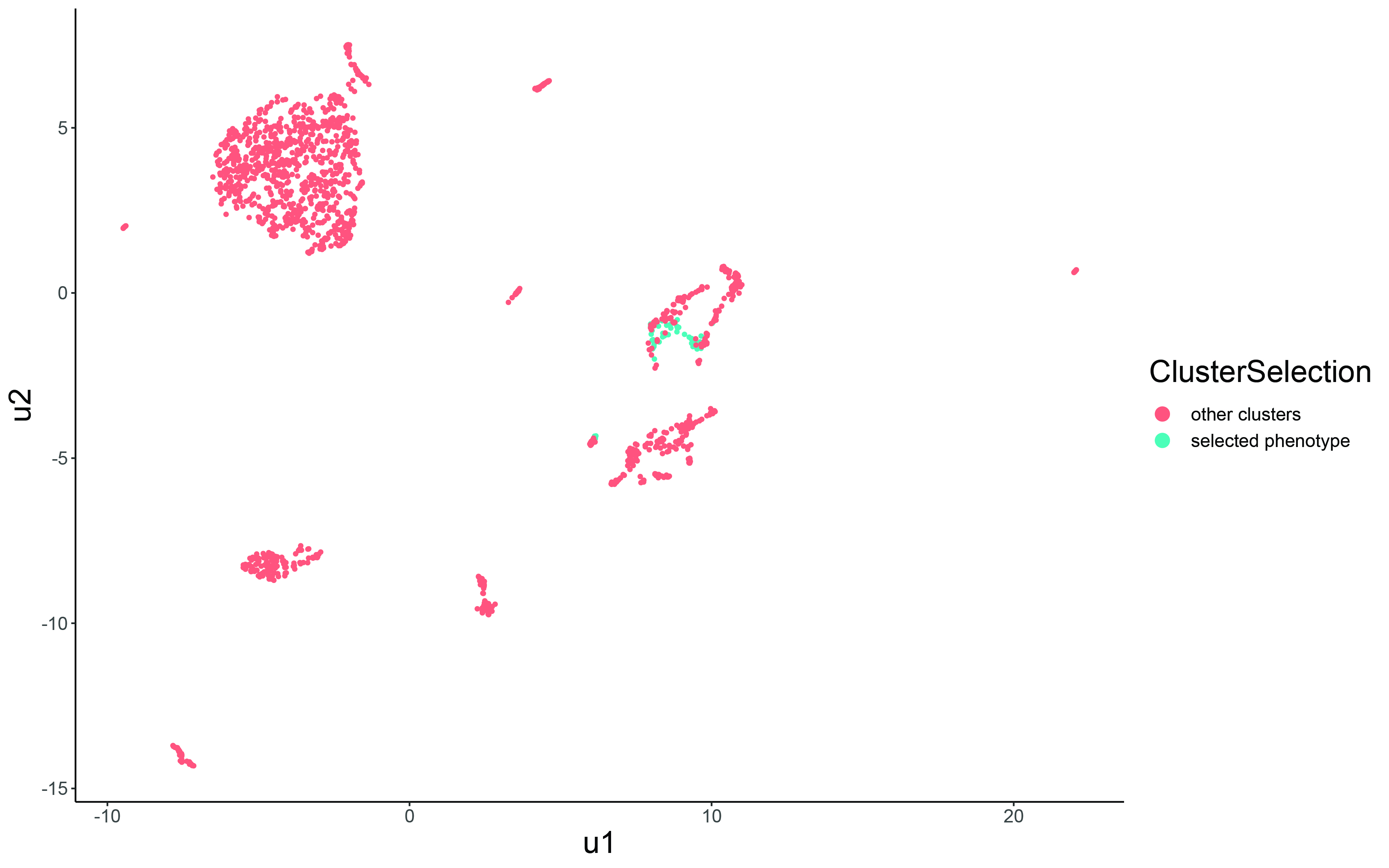

Supplement: btae595_Supplementary_Data [file btae595_supplementary_data.zip › CyCadas_FigureS3.jpg]

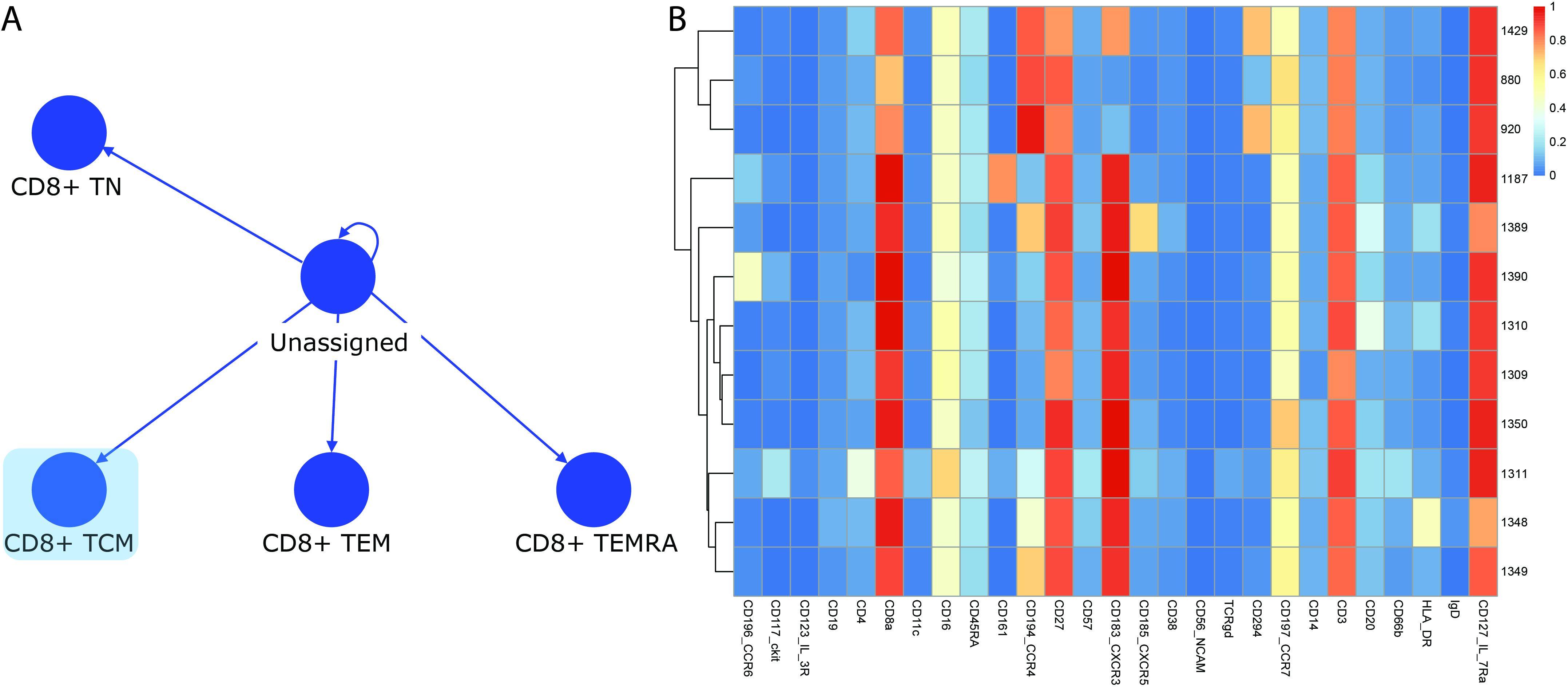

Supplement: btae595_Supplementary_Data [file btae595_supplementary_data.zip › CyCadas_FigureS2.jpg]

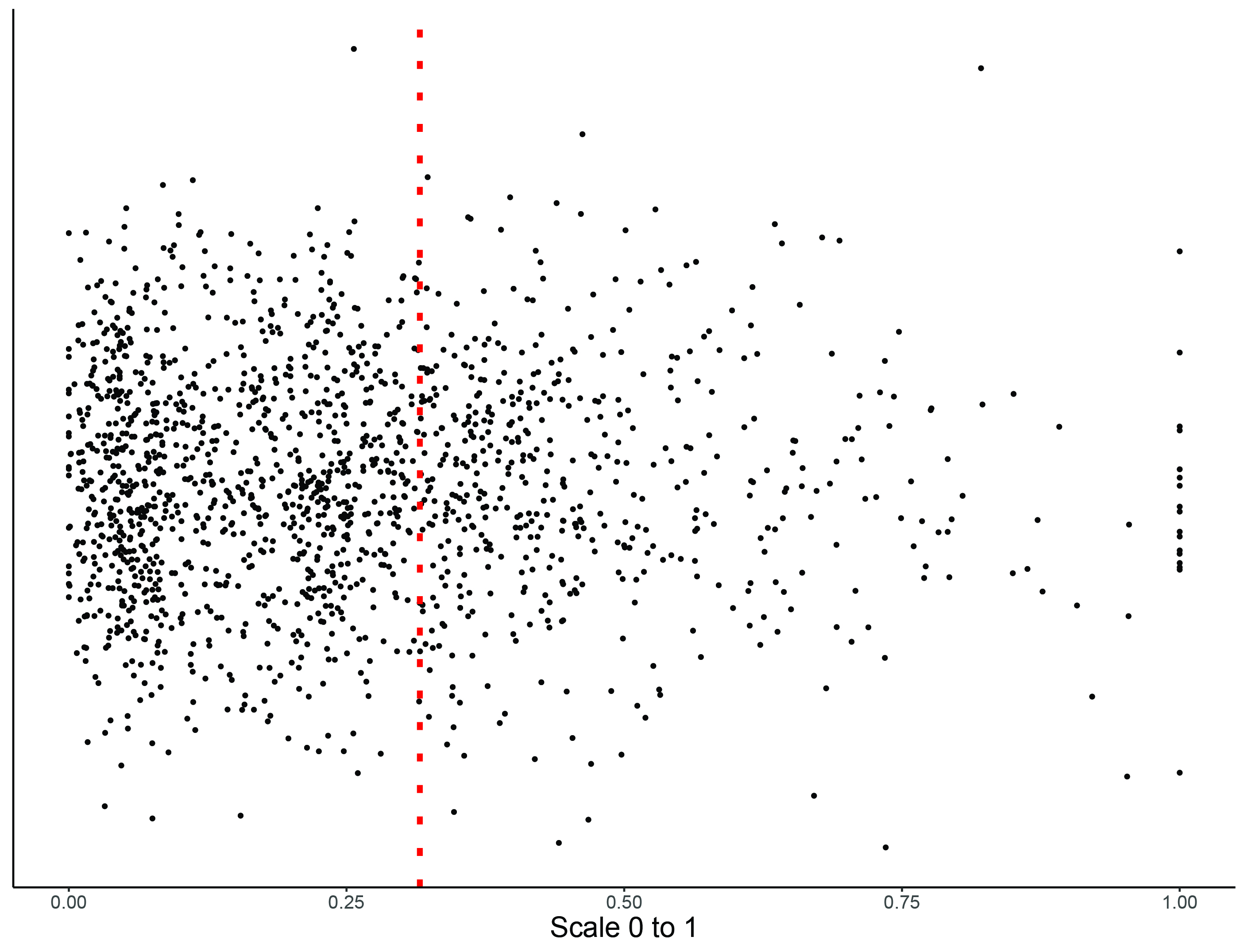

Supplement: btae595_Supplementary_Data [file btae595_supplementary_data.zip › CyCadas_FigureS1.jpg]

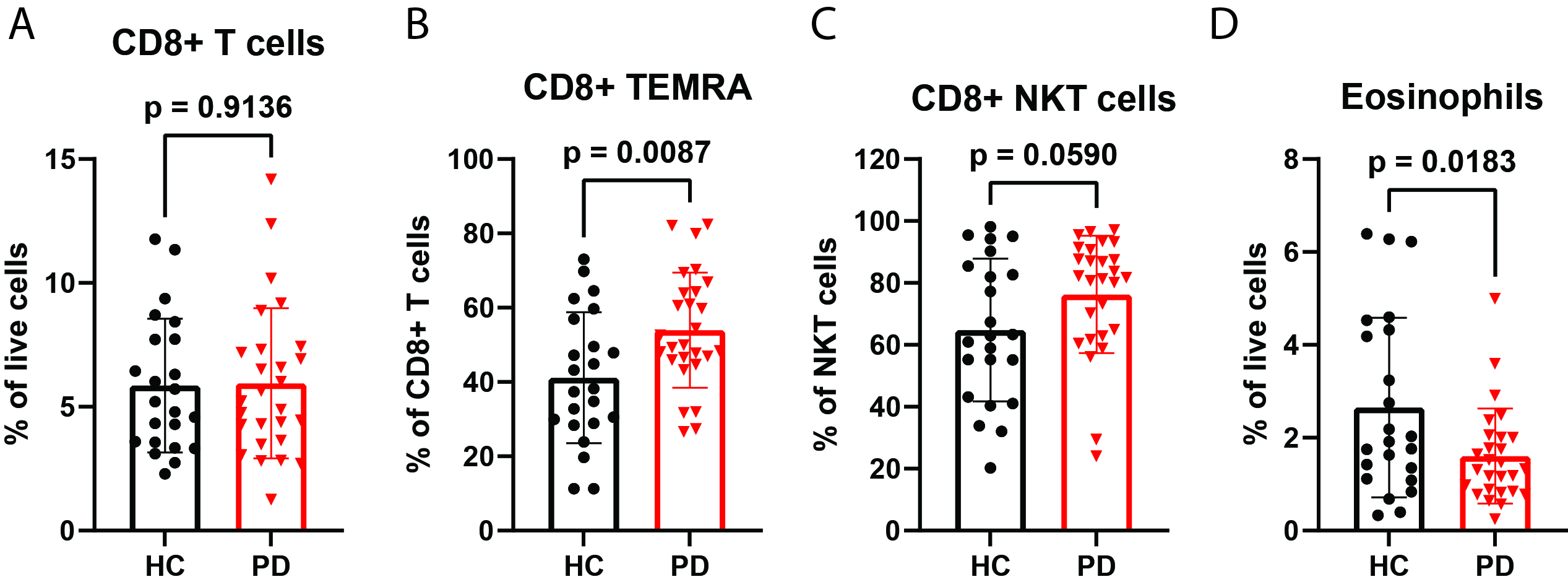

Supplement: btae595_Supplementary_Data [file btae595_supplementary_data.zip › CyCadas_FigureS4.jpg]
